# Use of Immune Profiling Panel to assess the immune response of septic patients for prediction of worsening as a composite endpoint

**DOI:** 10.1038/s41598-024-62202-z

**Published:** 2024-05-17

**Authors:** Estelle Peronnet, Gabriel Terraz, Elisabeth Cerrato, Katia Imhoff, Sophie Blein, Karen Brengel-Pesce, Maxime Bodinier, Aurore Fleurie, Thomas Rimmelé, Anne-Claire Lukaszewicz, Guillaume Monneret, Jean-François Llitjos

**Affiliations:** 1 Joint Research Unit HCL-bioMérieux, EA 7426 “Pathophysiology of Injury-Induced Immunosuppression” (Université Claude Bernard Lyon 1 – Hospices Civils de Lyon, bioMérieux), Lyon, France; 2 Open Innovation and Partnerships (OI&P), bioMérieux S.A., Marcy-l’Etoile, France; 3 EFOR, Champagne-au-Mont-d’Or, France; 4 Data Science, bioMérieux S.A., Marcy l’Etoile, France; 5 Anaesthesia and Critical Care Medicine Department, Hospices Civils de Lyon, Edouard Herriot Hospital, Lyon, France; 6 Immunology Laboratory, Edouard Herriot Hospital – Hospices Civils de Lyon, Lyon, France

**Keywords:** RNA, Infectious diseases, Immunology, Biomarkers

## Abstract

Sepsis induces intense, dynamic and heterogeneous host response modulations. Despite improvement of patient management, the risk of mortality and healthcare-associated infections remains high. Treatments to counterbalance immune response are under evaluation, but effective biomarkers are still lacking to perform patient stratification. The design of the present study was defined to alleviate the limitations of existing literature: we selected patients who survived the initial hyperinflammatory response and are still hospitalized at day 5–7 after ICU admission. Using the Immune Profiling Panel (IPP), a fully automated RT-qPCR multiplex prototype, we optimized a machine learning model combining the IPP gene expression levels for the identification of patients at high risk of worsening, a composite endpoint defined as death or secondary infection, within one week after sampling. This was done on 332 sepsis patients selected from two retrospective studies. The IPP model identified a high-risk group comprising 30% of patients, with a significant increased proportion of worsening events at day 28 compared to the low-risk group (49% vs. 28%, respectively). These preliminary results underline the potential clinical application of IPP for sepsis patient stratification in a personalized medicine perspective, that will be confirmed in a larger prospective multicenter study.

## Introduction

Sepsis is a complex, dynamic and heterogeneous syndrome, involving major changes in the immune response^1,2^. Owing to faster identification of sepsis and improvement in management of severe patients, patients now survive the early phase of sepsis and are now subjected to healthcare-associated infections (HAI)^3^. These HAI are associated with an impairment of the immune response and responsible for protracted length of stay and increased mortality^4^. Therefore, identification, prevention and treatment of nosocomial infections now appear as a cornerstone challenge in sepsis management^5^. However, despite decades of trials, treatments targeting the host response have failed to demonstrate benefits on clinical outcomes^6^. To circumvent this situation, it has been proposed to stratify patients on the basis of their immune status. In this approach, in which enrichment—either prognostic or predictive—is a key concept^7^, the objective is to identify patients that are at higher risk of disease-associated adverse outcome, or that could benefit the most from a new therapy. Numerous biomarkers have been proposed but so far none of these is effectively implemented in clinical routine to guide therapy in sepsis^8^.

Several parameters limit the use of biomarkers as stratification tools in current clinical practice. First, adoption is limited by technical factors such as poor standardization, automation, multiplexing capabilities, and the need of specific technical skills or sample preparation. Second, the heterogenous clinical performances reported in the literature do not allow clear guidelines on their use^9–11^. Indeed, in most studies, outcomes of interest are the prediction of death or HAI, considered independently, although death acts as a competing risk. Moreover, biomarkers from immune response seem to be regulated in the same way in patients who will either die or develop an HAI^12–14^. A composite outcome combining death and HAI could be an interesting alternative to this concern. Third, biomarker evaluation is often performed early after sepsis onset, whereas it could be more valuable to target patients who survived this extensive proinflammatory phase and display persistent signs of immune paralysis to evaluate immune-stimulating therapies, such as IFNγ, IL-7 or GM-CSF^15^. Finally, outcomes occurring within 28 days after markers measurement could be challenging to predict reliably. We postulated that the prediction of an outcome in a shorter timeframe could improve the performances.

In this setting, we decided to take advantage of a fully automated PCR multiplex molecular panel prototype measuring simultaneously the expression level of several mRNA markers of the host immune response from whole blood: the Immune Profiling Panel (IPP), that we previously evaluated in different clinical settings ^12,16,17^. The aim of this study is to evaluate the performances of the IPP prototype for the identification of a subgroup of high-risk sepsis patients, in a stratification perspective for a precision medicine approach. For this, we selected from two retrospective cohorts, patients who survived until day 5–7 after intensive care unit (ICU) admission and who did not already develop any HAI at this time. We then set up a classifier combining the expression level of the IPP gene set for the prediction of the occurrence of worsening, a composite outcome combining death and HAI, within 15 days after ICU admission.

## Materials and methods

### Patients

Sepsis patients were selected from the retrospective cohorts MIP Réa^18^ and REALISM^19,20^.

In the MIP Réa study (see^18^ for details on inclusion and exclusion criteria), sepsis patients aged > 18 years old with an expected length of stay > 2 days were enrolled between December 2009 and June 2011 in six French ICUs. Patients were followed up during all their ICU stay and were screened for ICU-acquired infection occurrence, according to the definitions used by the European Centre for Disease Control and Prevention^21^. The study protocol was approved by the local ethics committee (Centre d’Investigation Clinique IRB# 5044) which waived the need for informed consent from patients and/or next of kin (analyses performed on leftover blood), and is in accordance with the Helsinki Declaration of 1975. According to the French law at this date, patients or legal representatives were informed about the study and about their right to refuse to participate.

In the REALISM study (NCT02638779, see^19,20^ for details on inclusion and exclusion criteria), sepsis patients aged > 18 years old were enrolled in one ICU between December 2015 and June 2018. Patients were followed up during 28 days after study inclusion and were monitored for the occurrence of secondary infection, based on international guidelines^20^. Written informed consent was obtained from each patient. The study protocol was approved on December 3rd, 2015 by the Institutional Review Board (Comité de Protection des Personnes Sud-Est II) under number 2015-42-2 and is in accordance with the Helsinki Declaration of 1975.

In the present study, we focused the analysis on sepsis patients still in the hospital at day 5–7 after admission and without prior HAI at the time of sampling. The heterogeneity between the two cohorts in terms of severity, initial infection site, patient management or clinical outcomes (Table 1) led us to pool them and to divide the merged dataset into a train and a test sets (see Statistical analysis section).

**Table 1:** Patients baseline characteristics and management in the MIP Réa and REALISM cohorts.

Variable	All (n = 332)	MIP Réa (n = 245)	REALISM (n = 87)	*p* value
Baseline characteristics				
Gender, male	212 (64%)	155 (63%)	57 (66%)	0.806
Age, years	67 [57–77]	66 [55–76]	68 [59–78]	0.294
Type of admission				** < 0.001**
Medical	215 (%)	178 (73%)	37 (43%)	
Elective surgery	16 (%)	11 (4.5%)	5 (5.7%)	
Emergency surgery	101 (30%)	56 (23%)	45 (52%)	
Septic shock at inclusion	189 (57%)	128 (52%)	61 (70%)	**0.006**
Initial infection site				** < 0.001**
Respiratory	167 (50%)	144 (59%)	23 (26%)	
Abdominal	80 (24%)	45 (18%)	35 (40%)	
Others	85 (26%)	56 (23%)	29 (33%)	
Initial infection acquisition				0.158
Community acquired	222 (67%)	158 (65%)	64 (74%)	
Hospital acquired	110 (33%)	87 (36%)	23 (26%)	
Charlson score	4 [2–6]	4 [2–6]	4 [3–6]	0.643
SAPS II	55 [42–67]	58 [44–70]	46 [37–54]	** < 0.001**
SOFA at admission	9 [7–12]	9 [7–12]	9 [7–11]	0.101
Patient management				
Mechanical ventilation	223 (67%)	182 (74%)	41 (47%)	** < 0.001**
RRT	46 (14%)	26 (11%)	20 (23%)	**0.007**
Catecholamines	220 (66%)	154 (63%)	66 (76%)	**0.038**
Corticoids	70 (21%)	60 (25%)	10 (12%)	**0.016**
Outcomes at day 28				
Mortality	66 (20%)	58 (24%)	8 (9.2%)	**0.006**
HAI	65 (20%)	48 (20%)	17 (20%)	1.000

*p* values correspond to comparisons between the two cohorts and are bold when < 0.05.

### Immune Profiling Panel measurement

Peripheral whole blood was collected in PAXgene Blood RNA tubes at day 5–7 from ICU admission. PAXgene samples were stabilized for at least 2h after collection at room temperature and then frozen at − 80 °C following the manufacturer’s recommendations. RNA was then isolated as previously described^22,23^. RNA integrity was assessed using the RNA 6000 Nano Kit (Agilent Technologies, Santa Clara, CA, USA) on the Agilent 2100 Bioanalyzer (Agilent Technologies).

The determination of the mRNA expression level of host response markers was performed on the FilmArray® Torch Instrument (BioFire®, USA), as previously described^16,24^, by injecting 200 ng of isolated RNA in the Immune Profiling Panel prototype. Briefly, all freeze dried reagents are enclosed in a disposable pouch. After hydration and sample injection, the pouch is loaded in the FilmArray Torch Instrument, in which sample preparation, reverse transcription and nested PCR are performed. Among the 26 mRNAs available on IPP, we selected 11 markers as previously described^22^ (Supplementary Table S1). Normalized expression values were computed for each marker and used for the analyses.

### Outcomes definition

**Figure 1:**
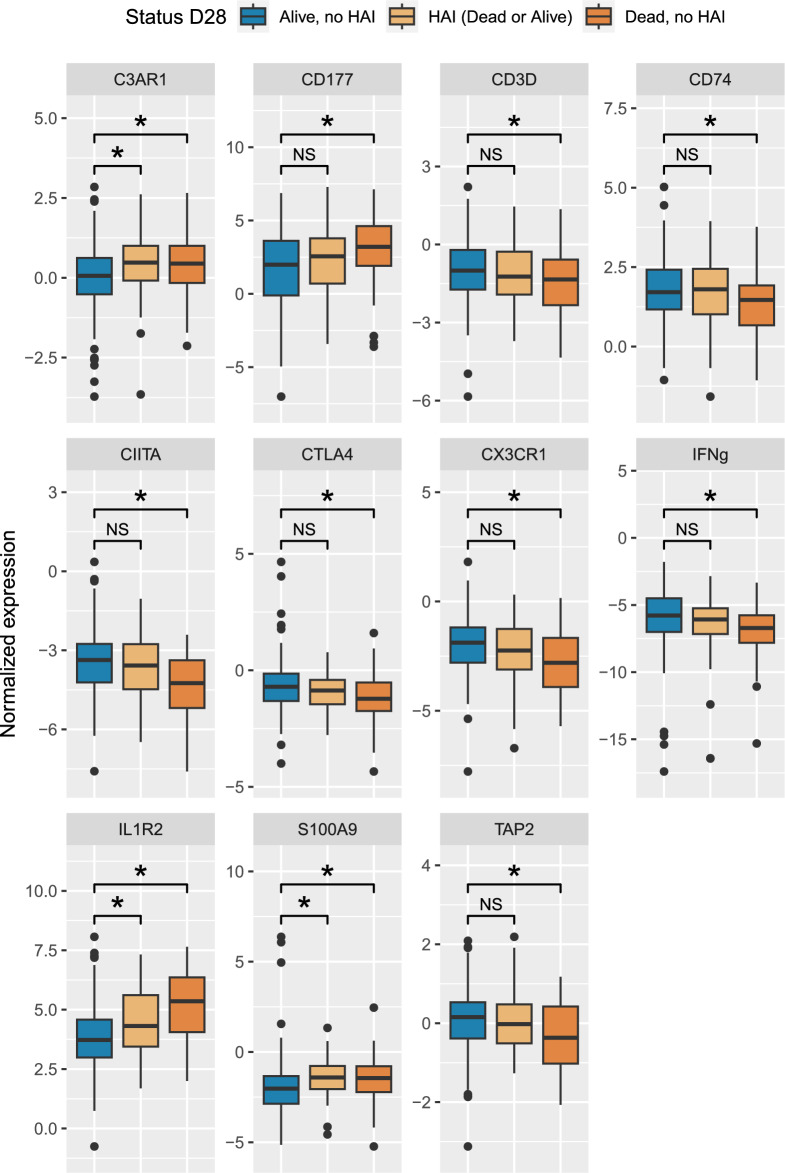
Boxplot representation of the expression level of the 11 IPP genes according to patient status at day 28: Alive, no HAI (n = 214), HAI (Dead or Alive) (n = 53) or Dead, no HAI (n = 65). Squared brackets report comparison between groups, either anova or pairwise Wilcoxon signed rank depending on the distribution. *p < 0.05. NS: non-significant difference.

For this study, patient follow-up period was censored at day 28 after study inclusion. Each clinical outcome was defined for two timeframes: until day 15 (close to sampling time-point) and until day 28. HAI was defined as a secondary infection that developed later 48h after ICU admission and while the patient was still in the ICU or in the hospital, until day 15 and day 28. In case of multiple HAI episodes, only the first episode was considered for the analysis. Worsening was defined as the occurrence of either HAI or death until day 15 or day 28. The use of a composite outcome allows to avoid risk competition between the two outcomes if considered separately. In addition, the IPP markers are regulated in the same way (either up- or down-regulated) in the HAI (dead or alive) group, or in the Dead no HAI group, compared to patients without event (Fig. 1). ICU stay, as well as treatments and organ support duration expressed in free days were also studied as outcomes (refer to Statistical analysis paragraph for free days computation).

### Statistical analysis

#### Clinical variables description and comparison

For continuous variables, medians, quartile 1 (Q1) and quartile 3 (Q3) were computed. Comparisons were done with either Student's t-test or Wilcoxon signed rank test depending on the distribution. For categorical variables, counts and percentages were computed and comparison were done with chi-squared test or Fisher’s exact test depending on the expected sample sizes. Reported p values were two-sided and a *p* value of < 0.05 was considered to indicate statistical significance. Free days scores^25^ were computed as follows: the patient gets one point for each day during the measurement period (15 or 28 days counted from the inclusion) where he is both alive and either free of administered cares or discharged from ICU, depending on the considered variable. A patient that dies during the measurement period receives a score of 0.

#### Predictive model development

A machine learning approach was implemented on the IPP gene set expression levels to predict worsening status (i.e., HAI occurrence or death; versus alive without HAI) within day 15 after ICU admission, based on a linear regression that used a space dimension reduction: the partial least square-discriminant analysis (PLS-DA) regression. This classification method can simultaneously manage many predictors, even if they are highly intercorrelated, which would hinder the identification of the features primarily associated with the outcome.

Pooled data from both cohorts were divided into a train set containing 217 samples (65%) to build the model and a test set containing the 115 remaining samples (35%) to assess the performance. The separation between the train and test set was balanced on worsening status, vital status and HAI status. The train set combining expression levels of the 11 IPP genes is centred and scaled. Means and standard-deviations values are set aside for future use with new data -like the test set- before making new predictions. The PLS-DA regression model was trained using repeated cross-validation 5X-tenfold for the prediction of worsening status. Synthetic minority over-sampling technique (SMOTE) was used to balance the training set between class^26^. Fine-tuning (number of components) was performed manually from one to the maximum number of components, selecting the best number of components according to the maximal mean area under the precision-recall curve (AUPRC) observed across repetitions. The final PLS-DA model was fit on the entire training set. Model performance was then evaluated on test set by calculating AUROC and its CI (bootstrap method, 2000 replicates), accuracy, sensitivity, specificity, positive predictive value, and negative predictive value. A descriptive analysis of patient outcomes according to worsening status defined by the IPP model was performed, using appropriate statistical tests. Analyses were performed using R software Version 3.6.2 (R Foundation for Statistical Computing, Vienna, Austria), with *caret* v6.0.84 and *pls* v2.7.3 packages for machine learning; *ggsurvfit* v0.2.0 and *survival* v3.4.0 for incidence plot.

## Results

### Patient description

Overall, 332 sepsis patients (245 from MIP Réa and 87 from REALISM) were still in the hospital on day 5–7 after ICU admission, without any diagnosed HAI at the time of sampling. Among them, 64 of 332 patients (19%) had a worsening status, defined as either death or HAI occurrence, at day 15. Their main baseline characteristics and management, as well as the clinical outcomes are described in Supplementary Table S2 and Supplementary Table S3, respectively. Median [Q1–Q3] age was 67 [57–77] years old and 64% of patients were male. At admission, the median SOFA score was 9 [7–12] and the median SAPS II was 55 [42–67]. The proportion of community-acquired initial infections was 67% of patients and lung was the primary site of infections in 50% of patients. At admission, 67% of patients were mechanically ventilated and 14% had renal replacement therapy. Globally, 20% (n = 66) of patients were deceased at day 28, independently of the occurrence of HAI. In total, 36% of patients experienced worsening until day 28: 16% died without any HAI and 20% developed an HAI. Among all HAI, pneumonia accounted for 51% of patients. We observed that 20% of patients who died had an HAI (13/66 patients). Among all patients that developed an HAI, 20% of them died (13/65). The median day 28 ICU free days was 16 [0–22] days.

The main clinical characteristics, management and outcomes were not different between the train and the test cohorts.

### Identification of a group of patients at high risk of worsening

The final trained model combining the expression level of the 11 IPP genes set was optimized for the identification of worsening status patients' class (who will develop an HAI and/or die within 15 days after ICU admission).

On the test set, 30% of patients were classified in the high-risk group. The demographic, comorbidity-related characteristics as well as patient management at admission were similar between the two groups (Table 2). Both severity scores (SAPS II and SOFA) were higher in the high-risk group. This model had an AUC of 0.69 [95% confidence interval 0.58–0.80] for the prediction of D15 worsening on the test set (Supplementary Table S4). This performance is slightly lower, while not statistically different, than the predictive ability of the SOFA score for the same endpoint (AUC = 0.73 [0.61–0.85]; *p* = 0.53). When used for the prediction of D28 worsening, the AUC of the IPP model was 0.63 [0.52–0.73] (Supplementary Table S4).

**Table 2:** Comparison of clinical characteristics at admission between the two groups identified by the IPP model.

Variable	Test set n = 115	High risk n = 35 (30%)	Low risk n = 80 (70%)	*p* value
Baseline characteristics				
Gender, male	74 (64%)	24 (69%)	50 (63%)	0.679
Age, years	64 [55–75]	62 [56–73]	66 [55–75]	0.371
Septic shock at inclusion	70 (61%)	24 (69%)	46 (58%)	0.362
Initial infection site				0.191
Respiratory	58 (50%)	17 (49%)	41 (51%)	
Abdominal	25 (22%)	11 (31%)	14 (18%)	
Others	32 (28%)	7 (20%)	25 (31%)	
Initial infection acquisition				**0.047**
Community acquired	76 (66%)	18 (51%)	58 (73%)	
Hospital acquired	39 (34%)	17 (49%)	22 (28%)	
Charlson score	4 [3–6]	4 [2–5]	4 [3–6]	0.128
SAPS II	55 [41–67]	60 [52–69]	48 [38–63]	**0.002**
SOFA	9 [7–11]	11 [9–13]	8 [6–10]	** < 0.001**
Patient management				
Mechanical ventilation	76 (66%)	26 (74%)	50 (63%)	0.310
RRT	14 (12%)	7 (20%)	7 (8.8%)	0.121
Catecholamines	76 (66%)	28 (80%)	48 (60%)	0.061
Corticoids	24 (21%)	11 (31%)	13 (16%)	0.111

*p* values correspond to comparisons between high-risk and low-risk groups. *p* values < 0.05 are bold.

**Figure 2:**
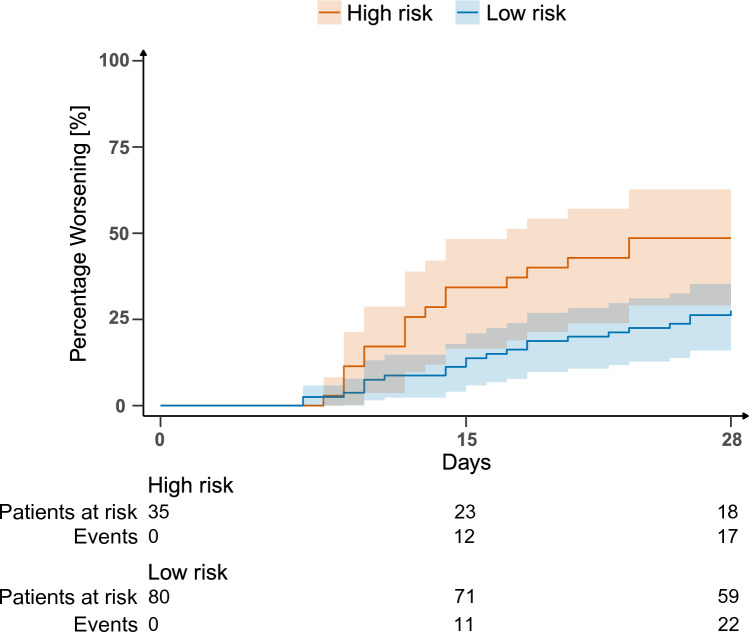
Cumulative incidence curves of worsening events in the test set, in the two groups of patients identified by the IPP model. Worsening is defined by the occurrence of death or healthcare-associated infection.

To illustrate the predictive enrichment capacity of IPP, besides the incidence of day 15 worsening, we compared several clinical outcomes at day 15 and day 28 in the groups of patients identified by the IPP model. As expected, the incidence of worsening was significantly higher in the high-risk group (Table 3, Fig. 2). At day 28, the proportion of patients that worsened was 1.75-fold higher in the high-risk group compared to the low-risk group. As expected, day 28 mortality was significantly higher in the high-risk group. Additionally, ICU free days, and all studied organ support and treatments free days were significantly lower in the high-risk group of patients identified by the IPP model. Moreover, as the IPP model performances seemed to be driven by mortality, we performed an analysis on patients who survived until day 28. The results revealed that the model was able to identify a group of patients who survived but had lower organ support free days (Supplementary Table S5). The proportion of patients developing an HAI and the HAI site were similar between the two groups (Table 3). Results were comparable for outcomes evaluated until day 15, despite less obvious differences.

**Table 3:** Enrichment in clinical outcomes at D15 and D28 in the two groups identified by the IPP model.

Variable	Test set n = 115	High risk n = 35 (30%)	Low risk n = 80 (70%)	*p* value
D15 outcomes				
Observed worsening D15	23 (20%)	12 (34%)	11 (14%)	**0.023**
Death without HAI	11 (10%)	8 (23%)	3 (4%)	
HAI	12 (10%)	4 (11%)	8 (10%)	
HAI D15 site				
Pulmonary	6 (50%)	2 (50%)	4 (50%)	0.717
Urinary	1 (8.3%)	0 (0.0%)	1 (13%)	
Bacteraemia	2 (17%)	1 (25%)	1 (13%)	
Abdominal	0 (0.0%)	0 (0.0%)	0 (0.0%)	
Catheter-related infection	2 (17%)	0 (0.0%)	2 (25%)	
Others	1 (8.3%)	1 (25.0%)	0 (0.0%)	
ICU Free days	3 [0–8]	0 [0–2]	6 [0–8]	** < 0.001**
RRT free days	15 [14–15]	15 [9–15]	15 [15–15]	** < 0.001**
Intubation free days	16 [12–23]	15 [9–20]	18 [13–23]	0.058
Catecholamines free days	12 [10–14]	10 [7–13]	12 [10–14]	**0.002**
D28 outcomes				
Observed worsening D28	39 (34%)	17 (49%)	22 (28%)	**0.047**
Death without HAI	15 (13%)	9 (26%)	6 (8%)	
HAI	24 (21%)	8 (23%)	16 (20%)	
HAI D28 site				
Pulmonary	14 (52%)	5 (56%)	9 (50%)	0.322
Urinary	4 (15%)	1 (11%)	3 (17%)	
Bacteraemia	3 (11%)	2 (22%)	1 (5.6%)	
Abdominal	1 (3.7%)	0 (0.0%)	1 (5.6%)	
Catheter-related infection	4 (15%)	0 (0.0%)	4 (22%)	
Others	1 (3.7%)	1 (11%)	0 (0.0%)	
ICU free days	16 [0–21]	4 [0–15]	19 [9–21]	** < 0.001**
RRT free days	28 [23–28]	23 [0–28]	28 [28–28]	** < 0.001**
Intubation free days	22 [9–26]	12 [0–23]	23 [17–27]	** < 0.001**
Catecholamines free days	24 [20–27]	22 [0–25]	25 [23–27]	** < 0.001**

*p* values correspond to comparisons between high-risk and low-risk groups. *p* values < 0.05 are bold.

## Discussion

Using a composite endpoint combining both HAI and mortality, we identified two groups of patients with different proportions of worsening using a machine-learned combination of gene’s expression levels. In the high-risk group, worsening occurred in almost 50% of patients at day 28 versus only 28% in the low-risk group. Patients from the high-risk group had also significantly longer length of ICU stay and mechanical ventilation.

Sepsis is a complex syndrome defined as a dysregulated host-response to infection responsible for organ dysfunction, with a high mortality rate^27^. Mortality in sepsis is highly influenced by several parameters such as individual genetic background, sepsis source, comorbidities and stages of illness ^28,29^. Given such heterogeneity, whole blood transcriptomic profiling, which consists in the analysis of the complete set of RNA transcripts produced by the circulating cells at a given time, appears as a promising tool. With the aim to identify patients with complicated clinical course early after ICU admission, studies have looked after gene signatures that could identify sub-groups of patients with poor clinical outcomes. For now, most of the studies have focused on mortality whereas HAI appear now as a cornerstone therapeutic challenge to improve the overall prognosis of ICU patients^10,30,31^. To do so, transcriptomic profiling can be used to stratify patients based on their risk of developing complications or their response to treatment. The recent advent of new devices such as the FilmArray IPP prototype, that measures simultaneously several host response gene expressions starting directly from whole blood, could allow to perform such assessment rapidly and efficiently ^12,17^.

In this study, despite a modest AUC, the IPP model showed a promising capability in prognostic enrichment in sepsis patients. Indeed, the proportion of patients that worsened within 28 days was 1.75-fold higher in the high-risk group compared to the low-risk group (49% vs. 28%, respectively), and 1.4-fold higher when compared to the global population without stratification (49% vs. 34%). Observational unsupervised studies previously published in the same research area reported comparable enrichments. For example, in the MARS study, mortality at day 28 was 1.62-fold higher in the MARS 1 endotype compared to all other endotypes (39% vs. 24%, respectively)^31^. In the discovery study reporting the identification of SRS endotypes, mortality was 27% in the SRS1 vs. 17% in the SRS2 endotype, i.e. a 1.59-fold increase^30^. The implementation of such stratification approach could help in clinical trial design and may contribute to facilitate therapeutic success, although, to the best of our knowledge, there is no guidelines providing the minimal performances to reach for such demonstration.

We observed an increased proportion of deceased patients in the high-risk group of patients when compared to low-risk patients, and also when compared to the global population. In our study, the pre-test probability to die without HAI at day 28 after ICU admission was 13%, whereas the post-test probability was 26%, i.e., twofold higher. For comparison, in the seminal publication of SEPSIS-3, the pre-test probability of lactate to predict mortality using the 2 mmol/L cut-off value was 30%, whereas the post-test probability was 36%, respectively, i.e. only 1.2-fold higher ^32^.

In our study, the model, although trained to identify deceased patients and those in whom HAI occurs, identifies a similar proportion of nosocomial infections in both high-risk and low-risk groups. This observation suggests that, between the two risk groups, the main driver of differences in gene expression level is death rather than HAI. Several hypotheses can be formulated to explain this observation. First, HAI are subjected to inter-observer variability and their diagnosis is subjective, even if adjudicated^33^. Second, most HAI are nosocomial pneumonia in both groups. Experimental studies have failed to report a distinctive transcriptomic systemic signature of ventilator-associated pneumonia in ICU patients^34,35^*.* Third, the occurrence of nosocomial infections, especially ventilator-associated pneumonia, is multifactorial with several risk factors, such as comorbidities, treatments and the duration of exposure to the invasive device. The weight of immune suppression remains to be determined. Fourth, there is a growing corpus of evidence suggesting that the attributable mortality of ICU-acquired infections is low^36^. Nevertheless, combining both HAI occurrence and mortality in one outcome remains clinically relevant since patients in the high-risk group harbour significantly longer durations of mechanical ventilation and higher rate of renal replacement therapy and catecholamine use. Moreover, the analysis performed on patient who were alive at day 28 revealed that, even if the performances of the model seem to be driven by mortality, IPP is able to identify a subgroup of patients with more severe outcomes, that might benefit from immunotherapy. In addition to previously published reports that used the IPP FilmArray prototype^12,17^, this new result provides clinically relevant information considering the competing risk between HAI and death.

This exploratory pilot study acknowledges several limitations. First, the retrospective design limits the conclusion that could be inferred from our results. The differences in the design of the two cohorts, as well as the variations in patient management guidelines over time, induced a great heterogeneity that we had to smooth by merging the two cohorts. Secondly, even if the results were obtained in a train/test approach, they need to be confirmed in a new independent multicenter study. Thirdly, as we performed gene expression measurement in whole blood, we cannot exclude that our model reflects differences in leukocyte subpopulations distribution rather than regulation of gene expression.

Using a multiplex molecular platform, we built a machine-learned model based on the combination of the expression of genes simultaneously assessed in blood, that identifies a subgroup of patients at high risk to worsen at day 28. These preliminary results are under evaluation in a large prospective ongoing international observational trial (IMPACCT, ISRCTN 11364482).

### Supplementary Information

Supplementary Tables.

## Data Availability

The datasets generated and/or analysed in the current study are available from the corresponding author upon reasonable request.
