# Supplementary material for: Use of Immune Profiling Panel to assess the immune response of septic patients for prediction of worsening as a composite endpoint
Source: Sci Rep. 2024 May 17;14:11305. doi: 10.1038/s41598-024-62202-z (PMC11101454; doi:10.1038/s41598-024-62202-z)
Supplement: Supplementary file 1 — Supplementary Tables. [file 41598_2024_62202_MOESM1_ESM.pdf]

Use of Immune Profiling Panel to assess the immune response of septic patients for prediction of worsening as a composite endpoint

Estelle Peronnet, Gabriel Terraz, Elisabeth Cerrato, Katia Imhoff, Sophie Blein, Karen Brengel-Pesce, Maxime Bodinier, Aurore Fleurie, Thomas Rimmelé, Anne-Claire Lukaszewicz, Guillaume Monneret, Jean-François Llitjos

Supplementary data

Supplementary Table S1 Prototype Immune Profiling Panel (IPP) gene set.

Supplementary Table S2 Patients baseline characteristics and management in the train and test sets.

Supplementary Table S3 Patients outcomes in the train and test sets

Supplementary Table S4 IPP model performances for the prediction of worsening at day 15 and day 28.

Supplementary Table S5 Enrichment in clinical outcomes at day 15 and day 28 in the two groups identified by the IPP model, on patients alive at D28.

Supplementary Table S1 Prototype Immune Profiling Panel (IPP) gene set.

Gene	Name
**C3AR1**	Complement C3a receptor 1
**CD177**	CD177 molecule
**CD3D**	CD3d molecule
**CD74**	CD74 molecule
**CIITA**	Class II major histocompatibility complex transactivator
**CTLA4**	Cytotoxic T-lymphocyte associated protein 4
**CX3CR1**	C-X3-C motif chemokine receptor 1
**IFNG**	Interferon gamma
**IL1R2**	Interleukin 1 receptor 2
**S100A9**	S100 calcium binding protein A9
**TAP2**	Transporter 2, ATP binding cassette subfamily B member

Supplementary Table S2 Patients baseline characteristics and management in the train and test sets.

Variable		All (n=332)	Train set (n=217)	Test set (n=115)	p value
**Cohort**					0.868
	MIP Réa	245 (74%)	159 (73%)	86 (75%)	
	REALISM	87 (26%)	58 (27%)	29 (25%)	
**Baseline characteristics**					
Gender, male		212 (64%)	138 (64%)	74 (64%)	0.987
Age, years		67 [57-77]	68 [57-78]	64 [55-75]	0.064
Septic shock at inclusion		189 (57%)	119 (55%)	70 (61%)	0.348
Initial infection site					0.689
	Respiratory	167 (50%)	109 (50%)	58 (50%)	
	Abdominal	80 (24%)	55 (25%)	25 (22%)	
	Others	85 (26%)	53 (24%)	32 (28%)	
Initial infection acquisition			217		0.922
	Community acquired	222 (67%)	146 (67%)	76 (66%)	
	Hospital acquired	110 (33%)	71 (33%)	39 (34%)	
Charlson score		4 [2-6]	4 [2-6]	4 [3-6]	0.487
SAPS II		55 [42-67]	54 [42-68]	55 [41-67]	0.614
SOFA at admission		9 [7-12]	9 [7-11]	9 [7-12]	0.173
**Patient management**					
Mechanical ventilation		223 (67%)	147 (68%)	76 (66%)	0.855
RRT		46 (14%)	32 (15%)	14 (12%)	0.632
Catecholamines		220 (66%)	144 (66%)	76 (66%)	1.000
Corticoids		70 (21%)	46 (21%)	24 (21%)	

p values correspond to comparisons between train and test sets, and are bold when <0.05.

Supplementary Table S3 Patients outcomes in the train and test sets

Variable	All (n=332)	Train set (n=217)	Test set (n=115)	p value
**D15 outcomes**				
Observed worsening D15	64 (19%)	41 (19%)	23 (20%)	0.923
Death without HAI	34 (10%)	23 (11%)	11 (10%)	
HAI	30 (9.0%)	18 (8.3%)	12 (10%)	
HAI D15 site				0.574
Pulmonary	15 (50%)	9 (50%)	6 (50%)	
Urinary	3 (10%)	2 (11%)	1 (8.3%)	
Bacteraemia	4 (13%)	2 (11%)	2 (17%)	
Abdominal	2 (6.7%)	2 (11%)	0 (0.0%)	
Catheter-related infection	2 (6.7%)	0 (0.0%)	2 (17%)	
Others	4 (13%)	3 (17%)	1 (8.3%)	
ICU Free days	2.50 [0-8]	2 [0-8]	3 [0-8]	0.519
RRT free days	15.0 [13.0-15.0]	15.0 [12.0-15.0]	15.0 [13.5-15.0]	0.317
Intubation free days	15.0 [12.0-23.0]	15.0 [12.0-23.0]	16.0 [12.0-23.0]	0.699
Catecholamines free days	12.0 [10.0-14.0]	12.00 [11.0-14.0]	12.00 [9.5-14.0]	0.209
**D28 outcomes**				
Observed worsening D28	118 (35%)	79 (36%)	39 (34%)	0.741
Death without HAI	65 (19%)	41 (19%)	24 (21%)	
HAI	53 (16%)	38 (17%)	15 (13%)	
HAI D28 site	65	24	41	0.548
Pulmonary	34 (52%)	13 (54%)	21 (51%)	
Urinary	11 (17%)	3 (13%)	8 (20%)	
Bacteraemia	6 (9.2%)	3 (13%)	3 (7.3%)	
Abdominal	5 (7.7%)	1 (4.2%)	4 (9.8%)	
Catheter-related infection	4 (6.2%)	3 (13%)	1 (2.4%)	
Others	5 (7.7%)	1 (4.2%)	4 (9.8%)	
ICU Free days	15 [0-21]	14 [0-21]	16 [0-21]	0.366
RRT free days	28.0 [13.0-28.0]	28.0 [3.0-28.0]	28.0 [22.5-28.0]	0.205
Intubation free days	21.5 [0.0-27.0]	21.0 [0.0-27.0]	22.0 [8.5-26.0]	0.631
Catecholamines free days	25.0 [19.8-27.0]	25.0 [18.0-27.0]	24.0 [20.0-27.0]	0.744

p values correspond to comparisons between train and test sets, and are bold when <0.05.

Supplementary Table S4 IPP model performances for the prediction of worsening at day 15 and day 28.

	Train set (n=217)	Test set (n=115)
**Performance at day 15**		
AUC	0.64 [0.55-0.74]	0.69 [0.58-0.80]
Accuracy	0.70 [0.63-0.75]	0.70 [0.62-0.78]
Sensitivity	0.46 [0.32-0.61]	0.52 [0.33-0.71]
Specificity	0.75 [0.68-0.81]	0.75 [0.65-0.83]
PPV	0.30 [0.20-0.42]	0.34 [0.21-0.51]
NPV	0.86 [0.79-0.90]	0.86 [0.77-0.92]
**Performance at day 28**		
AUC	0.67 [0.60-0.75]	0.63 [0.52-0.73]
Accuracy	0.69 [0.62-0.74]	0.65 [0.56-0.73]
Sensitivity	0.47 [0.36-0.58]	0.44 [0.29-0.59]
Specificity	0.81 [0.74-0.87]	0.76 [0.66-0.84]
PPV	0.59 [0.46-0.70]	0.49 [0.33-0.64]
NPV	0.73 [0.65-0.79]	0.72 [0.62-0.81]

Performances are reported with [95% confidence interval].

AUC: area under the receiver operating characteristic curve; PPV: positive predictive value; NPV: negative predictive value

Table S5 Enrichment in clinical outcomes at day 15 and day 28 in the two groups identified by the IPP model, on patients alive at D28.

Patients alive at day 28 only				
**Variable**	**Test set n=95**	**High risk n=23 (24%)**	**Low risk n=72 (76%)**	**p value**
**D15 outcomes**				
ICU Free days	5 [0-8]	0 [0-5.50]	6 [0.75-8]	**0.004**
RRT free days	15 [15-15]	15 [10-15]	15 [15-15]	**0.002**
Intubation free days	17 [13-23]	15 [10-21.5]	18 [13-24]	0.145
Catecholamines free days	12 [10-14]	11 [9.5-13]	12 [11-15]	**0.049**
**D28 outcomes**				
ICU free days	18 [10-21]	13 [5-18.50]	19 [145-21]	**0.006**
RRT free days	28 [28-28]	28 [23-28]	28 [28-28]	**0.002**
Intubation free days	23 [17.5-27]	18 [13.5-25.5]	24 [21-27]	0.061
Catecholamines free days	25 [23-27]	24 [22.5-26]	25 [24-28]	**0.049**

p values correspond to comparisons between high-risk and low-risk groups. p values <0.05 are bold
